# Hybrid Inductively Coupled Plasma and Computer-Controlled Optical Surfacing Polishing for Rapid Fabrication of Damage-Free Ultra-Smooth Surfaces

**DOI:** 10.3390/mi16091073

**Published:** 2025-09-22

**Authors:** Wei Li, Peiqi Jiao, Dawei Luo, Qiang Xin, Bin Fan, Xiang Wu, Bo Gao, Qiang Chen

**Affiliations:** 1 State Key Laboratory of Optical Field Manipulation Science and Technology, Chengdu 610209, China; liwei_ioe@163.com (W.L.); dtjiaopeiqi@163.com (P.J.);; 2 Institute of Optics and Electronics, Chinese Academy of Sciences, Chengdu 610209, China; 3 University of Chinese Academy of Sciences, Beijing 100049, China; 4 School of Optoelectronic Science and Engineering, University of Electronic Science and Technology of China, Chengdu 610054, China

**Keywords:** inductively coupled plasma, deposition layer, ultra-smooth surface, damage-free

## Abstract

The polymer deposition layer (PDL) formed during inductively coupled plasma (ICP) processing significantly limits the figuring accuracy and surface quality of fused silica optics. This study investigates the formation mechanism, composition, and evolution of the PDL under varying dwell times and proposes an innovative dwell time gradient strategy to suppress roughness deterioration. A significant disparity in hardness and elastic modulus between the deposition layer and the substrate is revealed, explaining its preferential removal and protective buffering effect in computer-controlled optical surfacing (CCOS). A hybrid ICP-CCOS polishing process was developed for processing a ϕ100 mm fused silica mirror. The results show that within 33 min, the surface graphic error RMS was significantly reduced from 58.006 nm to 12.111 nm, and within 90 min, the surface roughness was ultra-precisely reduced from Ra 1.719 nm to Ra 0.151 nm. The average processing efficiency was approximately 0.63 cm^2^/min. Critically, a damage-free, ultra-smooth surface without subsurface damage (SSD) was successfully achieved. This hybrid process enables the simultaneous optimization of figure accuracy and roughness, eliminating the need for iterative figuring cycles. It provides a novel theoretical framework for high-precision figuring and post-ICP polymer removal, advancing the efficient fabrication of high-performance optics.

## 1. Introduction

Fused silica components are known for their exceptional properties, such as high transmittance, excellent optical homogeneity, and outstanding corrosion resistance [1,2], which make them indispensable in high-performance optical systems operating under extreme conditions. In recent years, they have been extensively utilized in applications such as DUV lithography systems, laser confinement fusion reactors, space exploration instruments, and optical remote sensing technologies [3,4].

After grinding and shaping, post-polishing treatments are crucial for meeting stringent system requirements, including atomic-scale surface roughness and the elimination of SSD. However, achieving a balance between surface quality and processing efficiency remains a significant challenge [5]. Further research is necessary to resolve the opposing effects between form correction and roughness control, thereby improving the manufacturing efficiency of high-quality supersmooth surfaces.

As a typical hard–brittle material, fused silica is prone to surface and subsurface defects (e.g., scratches, cracks) when processed using conventional mechanical methods. Consequently, non-contact material removal techniques have gained prominence as effective alternatives. Currently, viable methods include magnetorheological finishing (MRF), ion beam figuring (IBF), and ICP polishing. MRF uses electromagnetic fields to manipulate magnetorheological fluids-composed of magnetic particles, base carrier fluids, and stabilizers-leveraging their rheological properties for polishing the workpiece [6]. This technique effectively suppresses plastic scratches and SSD associated with conventional polishing. However, residual base carrier fluid and magnetic particles within the MR fluid can adhere to surface microfeatures, potentially leading to surface contamination [7]. IBF enables high-precision processing of fused silica components but is highly dependent on initial surface conditions [8,9]. Additionally, its material removal mechanism can induce microstructural features at the microscopic or sub-micron scale on polished surfaces, complicating the balance between surface quality and processing efficiency [5]. Therefore, there is an urgent need to adopt a method that is highly adaptable to surface conditions, has high processing efficiency, and causes no damage to promote the manufacturing of high-quality surfaces.

ICP polishing, a non-contact technique that relies on chemical reactions for material removal [10], offers high material removal rates (MRR) [11]. Without mechanical contact or physical loading, it induces negligible surface damage (SD) or SSD [12], thus rendering it effective for rapid surface accuracy improvement. However, due to its material removal mechanism which relies on complex plasma-chemical etching reactions, incomplete reactions between active species and the workpiece surface may generate intermediate products that adhere to the surface [13,14,15,16]. In addition, impurity ions within the workpiece can be activated to form non-volatile compounds that remain solid on the surface, leading to a non-uniform deposition layer that partially covers the plasma-processed surface [17,18]. This phenomenon ultimately leads to surface roughness degradation [19].

To mitigate the impact of the deposition layer, Robert Heinke et al. applied laser treatment [20,21], which partially removed polymer deposits. However, this method induces residual stresses during cooling due to laser-thermal effects, and the precise removal of polymer deposits requires further investigation. The work by Su Xing, which utilized bonnet polishing on plasma-processed surfaces, maintained form accuracy but only reduced surface roughness from Ra 14.61 nm (post-ICP) to Ra 1.47 nm, failing to achieve sub-nanometer-level roughness [22]. Achieving simultaneous suppression of polymer deposition effects while preserving form accuracy and quality, and thereby enabling rapid convergence of both form error and surface roughness, remains a critical challenge. Therefore, this necessitates the development of a process capable of rapidly removing surface residue layers without introducing additional form errors or damage. While CCOS exhibits limited capability for high-precision figuring of fused silica, it demonstrates significant effectiveness in suppressing mid-to-high spatial frequency errors and reducing surface roughness on optical surfaces [23,24]. Through process optimization, the viscoelastic properties of pitch polishing pads enable conformal contact with the workpiece surface. This approach shows promise as an effective method for eliminating post-plasma-processing residue layers on fused silica surfaces.

Therefore, this study initially examined the causes behind the formation of PDL during plasma processing. By analyzing the compositional characteristics of plasma jets and the elements within the PDL, this study investigated the changes in the state of these layers under varying dwell times and proposed a method for dividing dwell time into gradients. The continuous dwell time gradient for a single point was divided into two segments: 60% and 20%. While ensuring processing efficiency and accuracy, the deterioration of surface roughness was suppressed.

Next, the differences in mechanical properties between the deposition layer and the fused silica matrix were analyzed. Due to the low hardness and elastic modulus of the PDL, it is preferentially removed during the CCOS process. Meanwhile, the presence of the deposit layer provides a buffering effect for the embedding of abrasive particles in the asphalt surface, preventing the scratching of the surface by large abrasive particles, thus reducing mechanical damage and defects. This resulted in an improvement in surface quality.

Finally, the ICP-CCOS method was employed to perform high-performance processing on a fused silica mirror surface with a diameter of 100 mm. By applying ICP-CCOS technology for 124 min, the surface accuracy of the workpiece improved from RMS 58.006 nm to RMS 12.111 nm. The surface roughness Ra was reduced from 1.719 nm to 0.151 nm, and a defect-free surface without SSD was obtained. The results demonstrate that the ICP-CCOS composite polishing method efficiently achieves the convergence of surface shape error and roughness of optical components, resulting in a non-destructive, super-smooth surface. The research findings offer valuable insights for the high-precision shaping of fused silica and the effective removal of deposition layers after ICP processing. This is crucial for the efficient, high-quality manufacturing and application of non-destructive, ultra-smooth surface optical components.

## 2. Materials and Experimental Methods

### 2.1. Materials

The fused silica specimen is made of ϕ100 mm × 10 mm fused silica glass (JGS1), with an initial face shape accuracy of RMS 50.008 nm and a surface roughness of Ra 0.487 nm. The gases utilized in ICP include 99.999% argon (Ar) and carbon tetrafluoride (CF_4_), sourced from Dalian Datai Gas (Dalian) Co., Ltd. in China. A cerium oxide polishing solution is employed in CCOS, with an average particle size of approximately 20 nm for the CeO_2_, which was procured from Beasley New Materials (Suzhou) Co., Ltd. in China. The additive dodecylbenzene sulfonic acid (DBSA), supplied by Henan Zanyu Technology (Hebi) Co., Ltd. in China, has a purity exceeding 96%. Both the processing equipment for ICP and CMP have been independently developed.

### 2.2. Methods

**Figure 1:**
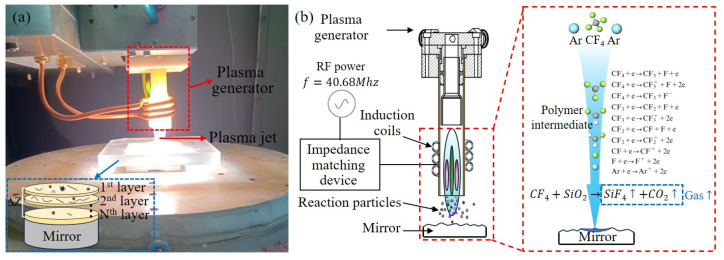
The equipment and processing principle of ICP. (**a**) Experimental equipment; (**b**) Schematic diagram of plasma generation and function.

Plasma processing utilizes plasma to excite reactive gases, transforming them into active particles. The active particles, driven by gas jets, are transported to the workpiece surface, where they undergo chemical reactions with the surface material, generating volatile products and thereby achieving material removal [25]. In this study, 40.68 MHz inductively coupled radio frequency power was supplied to the plasma through the load coil via the impedance matching box (PSG-III, Rishige, Changzhou, China). The schematic diagram of the ICP process for the Ar/CF_4_ mixture is shown in Figure 1. The torch consists of three coaxial quartz tubes, through which two tangential streams of ultra-pure argon plasma gas pass, one through the outer gap and the other through the inner gap. Meanwhile, the reactant gas CF_4_ is introduced into the plasma via the inner tube. Subsequently, radio frequency power is applied to the load coil. The gas is then ignited by the free electrons generated by the high-voltage spark. Finally, the CF_4_ molecules dissociate under electron bombardment, generating various active species such as CF_3_, CF_2_, CF, and F [14]. Inductively coupled plasma generates active free radicals, which are transported by plasma jets and diffuse onto the substrate’s surface, where they interact with surface materials. For example, F free radicals exhibit high reactivity, combining with Si atoms in SiO_2_ to break the Si-O bond and produce gaseous silicon tetrafluoride (SiF_4_) under standard temperature and pressure conditions. The main chemical reaction can be represented by the following equation:

(1) $${\mathrm{CF}}_{4}+{\mathrm{SiO}}_{2}\to {\mathrm{SiF}}_{4}↑+{\mathrm{CO}}_{2}↑$$

As a gaseous jet-based technique, plasma chemical processing establishes no physical contact with the workpiece. Consequently, it imposes no mechanical forces on the surface, thereby eliminating SD typically associated with mechanical machining [19,26]. Furthermore, studies have demonstrated its ability to remediate pre-existing SSD caused by mechanical grinding, ultimately resulting in damage-free surfaces.

Surface form accuracy before and after ICP processing was measured using a laser interferometer (ZYGO Corporation GPI XP/D, Middlefield, CT, USA). Surface topography and roughness of polymer deposits were quantified using white-light interferometry (Bruker Nano Surfaces NPFLEX-1000, Santa Clara, CA, USA), while atomic force microscopy (AFM, Bruker Dimension Icon, Santa Barbara, CA, USA) provided complementary measurements of final surface morphology and nanoscale roughness. Chemical composition of polymer deposits was analyzed using X-ray photoelectron spectroscopy (XPS) with a standard Al Kα source (ULVAC PHI 5000 VersaProbe, Chigasaki, Japan). Nanomechanical properties of the deposited regions were evaluated using nanoindentation (Agilent G200, Santa Clara, CA, USA). High-resolution scanning electron microscopy (SEM, Zeiss Sigma300, Oberkochen, Germany) was employed to characterize the morphology of polymer deposits. For subsurface analysis, cross-sectional specimens were prepared using focused ion beam (FIB) milling, followed by transfer to a transmission electron microscope (TEM, Thermo Fisher Scientific Talos F200X, Hillsboro, FL, USA), enabling the observation of deposition layer and SSD distribution, with compositional verification via energy-dispersive X-ray spectroscopy (EDX).

## 3. Results and Discussion

### 3.1. Characterization and Formation Mechanism of PDL

**Figure 2:**
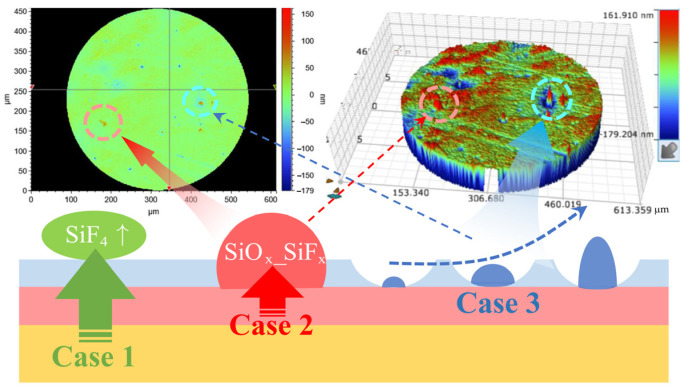
The manifestation forms of deposition layer.

**Figure 3:**
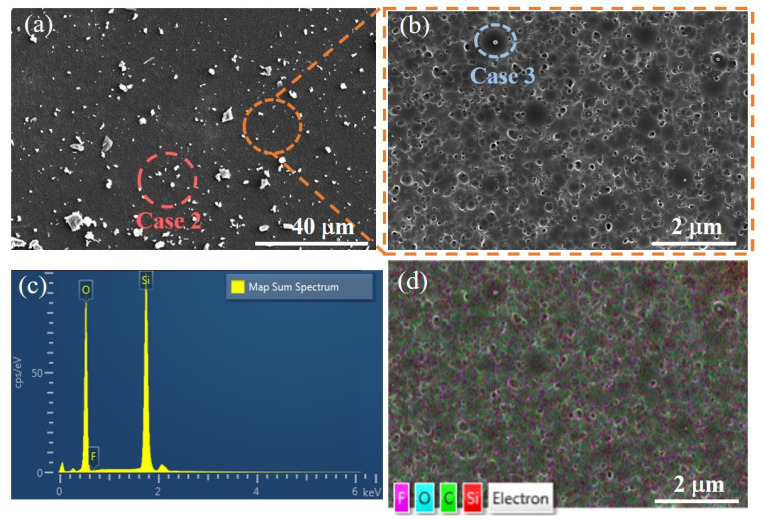
SEM measurement results of the deposition layer area. (**a**) Morphology and distribution of case 2 in the deposition layer region; (**b**) Morphology and distribution of case 3 in the deposition layer region; (**c**) Elemental composition of the deposition layer region; (**d**) Element distribution in the deposition layer area.

Spot tests for characterizing the removal function were conducted using stable ICP parameters (as Table 1). Post-processing surfaces were characterized using SEM and white-light interferometry. The material removal during the ICP etching process is facilitated by fluorine-containing ionizable substances. As illustrated in Equations (2) and (3), the active F atoms in a physically adsorbed state directly react with the silicon dangling bonds on the surface of SiO_2_ to form Si-F bonds. Subsequently, additional F atoms attack the fluorinated surface structure, ultimately resulting in the complete detachment of Si and the formation of gaseous Si-F_4_, along with the release of oxygen gas (O_2_). The dangling bonds are reformed after the fused silica substrate has detached from the silicon. During etching, surface reactions yield fluorocarbon (CF_x_) films and induce SiF_x_ (x = 1, 2, 3) and Si-O-C bonding at the CF_x_/SiO_2_ interface [27]. The multistep reaction pathway involves the adsorption, ionization, and dissociation of species, making the reaction equilibrium during etching inherently incomplete. On the one hand, as illustrated in Equations (4) and (5), gaseous CF_x_ (x = 1–3) groups are adsorbed onto the dangling bonds on the surface of SiO_2_. Subsequently, F atoms attack the adsorbed CF_x_ groups, increasing their degree of fluorination. This process can lead to the formation of a fluorocarbon polymer intermediate (CF_x_) and the release of gaseous CF_x+1_ species, such as CF_4_. Alternatively, the polymerization process described in Equations (6) and (7) occurs, wherein the adsorbed CF_x_ groups polymerize on the surface to form a polymer (P). F atoms or CF_x_ groups continue to adsorb onto the existing polymer, facilitating its further growth. On the other hand, SiF_x_ (x = 1, 2, 3) is unstable in atmospheric conditions and tends to convert into SiF_4_ gas. However, the presence of impurity metal elements in fused silica, along with the difficulty of removing metal oxides from the workpiece surface in gaseous form, may result in their accumulation on the surface as metal silicates or metal oxides. This accumulation contributes to the formation of surface deposits. Key reactions are as follows [28,29,30,31,32]:

(2) $$O_{2}-{\mathrm{Si}}^{*}\left(s\right)+2F\left(p\right)\to O_{2}-\mathrm{Si}-F_{2}\left(s\right)$$

(3) $$O_{2}-\mathrm{Si}-F_{2}\left(s\right)+2F\left(pa\right)\to 2O_{2}-{\mathrm{Si}}^{*}+\mathrm{Si}-F_{4}\left(g\right)+O_{2}\left(g\right)$$

(4) $$\mathrm{Si}-{O_{2}}^{*}+\;{\mathrm{CF}}_{x}\left(g\right)\to \mathrm{Si}-O_{2}-{\mathrm{CF}}_{x}\left(s\right)$$

(5) $$\mathrm{Si}-O_{2}-{\mathrm{CF}}_{x}\left(s\right)+F\left(g\right)\to \mathrm{Si}-O_{2}+{\mathrm{CF}}_{x+1}\left(g\right)$$

(6) $$\mathrm{Si}-O_{2}-{\mathrm{CF}}_{x}\left(s\right)\to P$$

(7) $$P-F\left(s/P\right)\to P;\;{P-\mathrm{CF}}_{x}\left(s/P\right)\to P$$

where ‘*’ represents dangling bonds, s represents chemically adsorbed atoms or groups of atoms, ‘pa’ represents physically adsorbed atoms, ‘g’ represents the gaseous state, ‘P’ represents the polymer, and ‘s/P’ represents chemically adsorbed atoms or groups of atoms on the polymer surface. The existence forms of deposition layers mainly include peak-shaped protrusions and convex peaks within etching pits, as shown in case 2 and case 3 in Figure 2, which lead to the deterioration of surface roughness after etching [15,33]. However, due to the coexistence of both base material etching and polymer deposition during processing, the thickness of the PDL does not increase indefinitely but instead reaches a stable state [27,34,35]. Nevertheless, to enhance the subsequent surface quality, the deposition layer should be minimized in thickness. To further investigate the cause of the roughness deterioration, the element was subjected to a single-point etching for 3 min, The PDL area on the surface was examined using SEM at high magnifications. The surface conditions are depicted in Figure 3a,b. Two forms of cases, as shown in Figure 2, were observed, and EDS analysis was performed on the area shown in Figure 3b to generate Figure 3c,d. The primary elemental composition on the surface consists of Si, O, F, and C, and the elemental distribution is shown in Figure 3d. However, the exact composition of the polymer deposition still requires further analysis.

**Figure 4:**
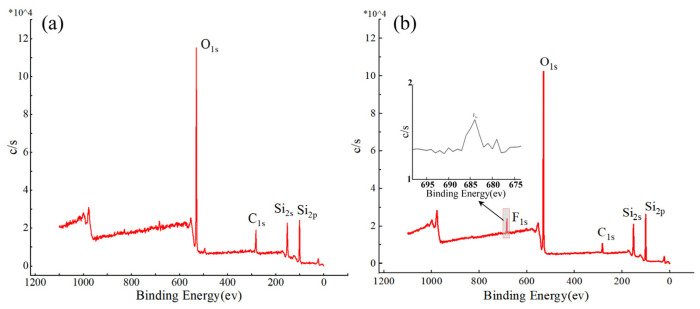
The full spectral lines of XPS in the initial surface and polymer deposition area: (**a**) initial surface; (**b**) deposition layer area.

To further analyze the composition of these polymer deposits, we employed XPS for their analysis. Firstly, XPS detection is performed on the surface of the workpiece before plasma processing, serving as a reference benchmark for the changes in the surface’s chemical composition after processing. The full spectral line analysis results for the elements on the surface of fused silica before and after ICP processing are shown in Figure 4a,b. Based on the XPS element spectral line fitting results, it can be observed that, in addition to the three elements (Si, O, and C) present on the surface before processing, a peak corresponding to the F element has emerged on the surface of fused silica after ICP processing.

**Figure 5:**
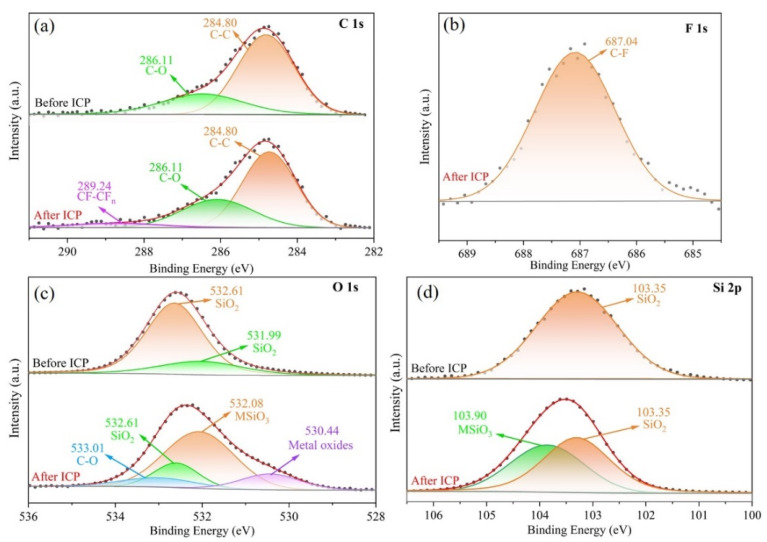
XPS analysis was conducted on the elements C 1 s (**a**), F 1 s (**b**), O 1 s (**c**), and Si 2p (**d**) before and after ICP.

Further analysis of the chemical bonds of the four elements yielded high-resolution spectra, as illustrated in Figure 5a. Before ICP processing, the peak binding energies of the C 1 s element were 284.8 eV and 286.11 eV, which resulted from contamination introduced by the sample’s exposure to air during the detection process [36,37]. However, after ICP treatment, the C 1 s spectrum exhibited a new peak with a binding energy of 289.24 eV. Through analysis and comparison, it was determined that this peak corresponds to CF-CF_n_ [38], while the 687.04 eV peak of F 1 s corresponds to C-F [39], as shown in Figure 5b. Therefore, fluorocarbons are present in the deposition layer. As depicted in Figure 5c, the peak binding energy of O 1 s increased from the original 532.61 eV and 531.99 eV [40,41], corresponding to SiO_2_, to 533.01 eV for C-O [42], along with silicates (MSiO_3_) and metal oxides associated with the metal elements in fused silica, which correspond to 532.08 eV and 530.44 eV [43], respectively. Additionally, following Si 2p ICP processing, apart from the 103.35 eV peak corresponding to SiO_2_ [39], a newly added 103.90 eV peak corresponds to MSiO_3_ [44,45]. This indicates that in the deposited layer, in addition to CF-CF_n_, the metal elements in fused silica cannot escape as gas during ICP processing. As the amount of SiO_2_ removed increases, the metal elements undergo enrichment on the surface and exist in the deposition layer in the form of MSiO_3_ and metal oxides.

**Figure 6:**
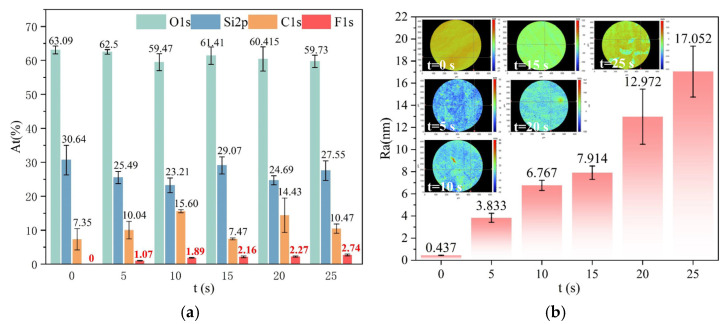
The content and roughness changes of each element in the processed area after different dwell times. (**a**) Changes and comparisons of the contents of each element; (**b**) The roughness varies with the dwell time.

Under constant process parameters (Table 2), the duration of plasma exposure has a significant effect on the fluorocarbon deposition process. In order to quantify the relationship between fluoride concentration and exposure time, five adjacent regions on a single substrate were subjected to processing with incrementally increasing durations (0, 5, 10, 15, 20, 25 s). The relative concentrations of O, Si, C, and F were measured using XPS, as illustrated in Figure 6a. The measurements were conducted with a sampling depth of 5 nm and a spot size of 400 μm. Among these, the fluorine content is primarily correlated with the deposition layer and the resulting deterioration in surface roughness. The figure shows that the fluorine content is positively correlated with the dwell time. Figure 6b presents the surface roughness results corresponding to different dwell times. The figure indicates that the degree of surface roughness deterioration is positively correlated with dwell time, due to varying processing durations. Longer dwell times at a single point result in greater surface roughness deterioration, following the formation of the deposition layer by ICP.

**Figure 7:**
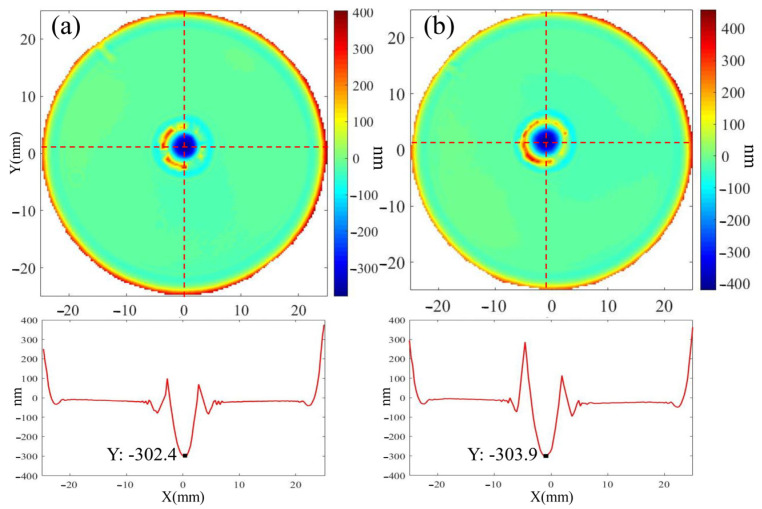
Comparison of results after multiple cumulative processing and a single continuous processing: (**a**) Peak removal amount after multiple cumulative processing; (**b**) Peak removal amount after a single continuous processing.

To compare the effects of single continuous processing versus multiple cumulative removals on material removal and surface roughness, two fused silica elements with an initial roughness of Ra 0.451 nm were selected, with the total dwell time at a single point being the same in both cases. Sample A was processed five times under the same processing parameters, with each processing duration being 2 s, as shown in Table 3. The total removal time for sample A was 10 s. Sample B was processed only once for a duration of 10 s. The removal depth and surface roughness of the processed area were measured. The results of the surface shape detection, including the contour removed during processing, are shown in Figure 7. The maximum material removal in the central area can be detected. When sample A undergoes cumulative processing for 10 s, the maximum removal depth reaches 302.4 nm. When sample B is processed for 10 s, the maximum removal depth is 303.9 nm. The results show that under the same total dwell time, both single continuous processing and multiple cumulative removals have minimal impact on material removal depth.

**Figure 8:**
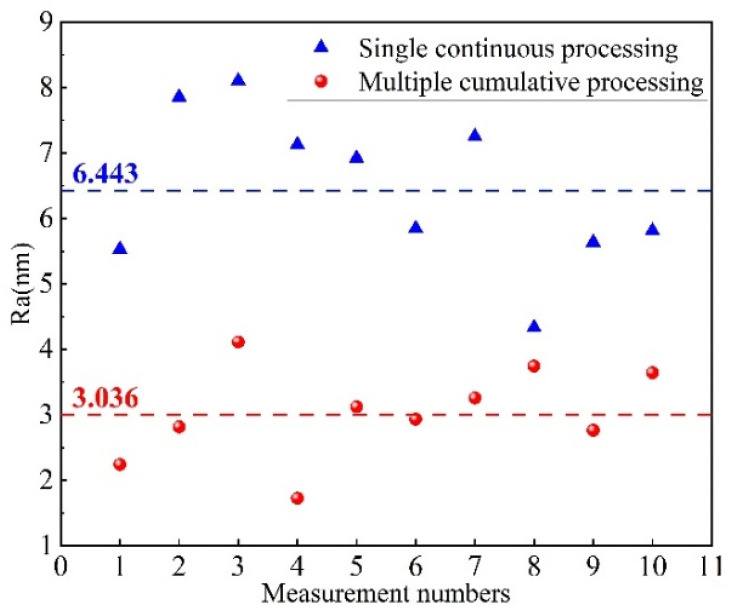
Comparison of surface roughness after multiple cumulative processing and a single continuous processing.

Figure 8 presents the roughness detection results obtained from 10 measurements at different positions of two samples after processing. The results indicate that the average roughness, Ra, of sample A after multiple cumulative processing steps is 3.036 nm, which is significantly lower than the average roughness, Ra, of sample B, which is 6.443 nm. The main reason is as follows: On the one hand, during the prolonged single ICP processing, the temperature at the processing point gradually increases, resulting in an accelerated reaction rate. In parallel, the adsorption reaction intensifies, leading to a severe deposition of the polymer. On the other hand, continuous ICP processing leads to the formation of a continuously thickening PDL at the center, which shields the effect of fluorine free radicals [18]. Following repeated removal, the ICP chemical reaction preferentially interacts with the intermediate products (CF_x_/Si_y_) deposited on the surface, in conjunction with the aforementioned Equations (5) and (7). Due to its inherent instability, SiF_3_ undergoes further fluorination under plasma action, resulting in the formation of SiF_4_, which subsequently volatilizes, as illustrated in Equation (8). Additionally, the CF_x_ species connected by suspended bonds on the surface of SiO_2_, as well as the CF_x_ present in the formed polymer (P), may also undergo further fluorination, as illustrated in Equations (9) and (10), generating CF_4_ gas that volatilizes and detaches from the surface:

(8) $${\mathrm{SiF}}_{3}\left(s\right)+F\left(p\right)\to {\mathrm{SiF}}_{4}\left(g\right)$$

(9) $$\mathrm{Si}-O_{2}-{\mathrm{CF}}_{3}\left(s\right)+F\left(p\right)\to {\mathrm{CF}}_{4}\left(g\right)+\mathrm{Si}-O_{2}$$

(10) $$P-{\mathrm{CF}}_{x}\left(s\right)+\left(4-x\right)F\left(p\right)\to {\mathrm{CF}}_{4}\left(g\right)+P$$

### 3.2. ICP Enhances the Surface Shape Accuracy of Optical Components

Based on the experimental results from the previous section, multiple removal cycles, under the same material removal, can mitigate the deterioration of surface roughness caused by the PDL and reduce the impact of thermal accumulation on the stability of the removal process [46]. Therefore, when plasma is used for shaping, surface quality after processing can be enhanced by employing multiple cumulative processing steps. In this context, a resident time gradient partitioning method is proposed for surface shape refinement. To ensure both speed and accuracy in surface shape convergence, the processing program, initially achieving 80% convergence, is optimized as follows: In the first shaping step, the surface shape error convergence ratio is set at 60% to rapidly eliminate high-point surface errors. In the second shaping step, the surface shape error convergence ratio is set at 20% to further correct minor surface shape errors. With the overall material removal and processing time remaining essentially unchanged, efficient and high-quality shaping of the workpiece surface is achieved.

**Figure 9:**
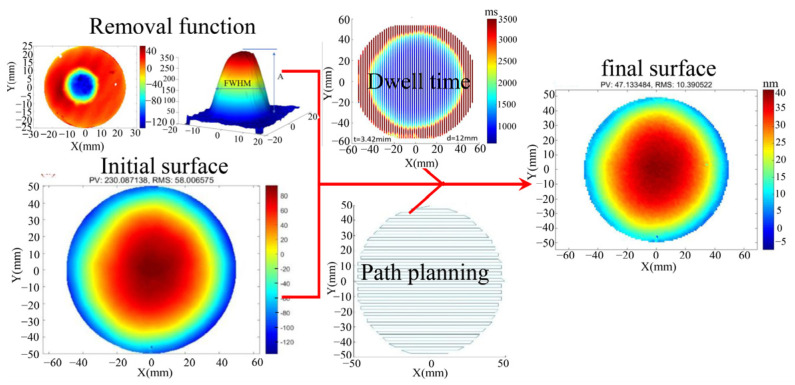
Schematic diagram of the principle of ICP improving surface shape accuracy.

**Figure 10:**
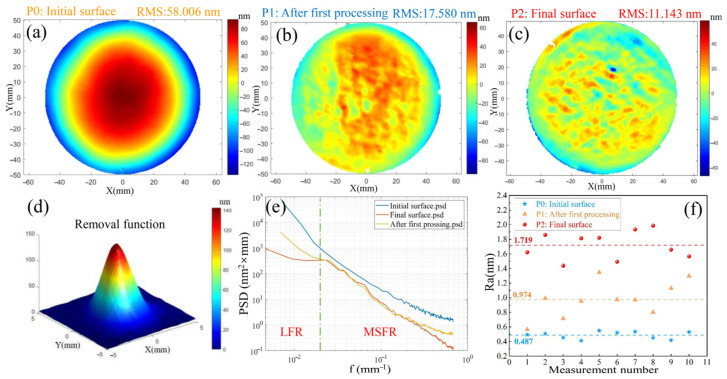
The ICP precision modification process using the resident time gradient division method: (**a**) The initial surface shape of the workpiece; (**b**) The surface shape after the first processing; (**c**) The final surface shape after the second processing; (**d**) Removal function of the processing procedure; (**e**) Comparison of PSD curves of the surface shape throughout the entire processing procedure; (**f**) Changes in the surface roughness of the workpiece.

Based on the principle that material removal equals the convolution of dwell time and the removal function, the surface shape convergence rates are set at 60% and 20%, respectively, to generate grating trajectories. The modification principle is illustrated in Figure 9, with material removal being controlled through the adjustment of single-point resident time. The ICP contour modification experiment was conducted according to the process parameters outlined in Table 4. The removal function is presented in Figure 10d. The width of the removed contour is approximately 4 mm, with a peak removal rate of approximately 1.7 μm/min. It not only offers high resolution, meeting the requirements for high-precision contour modification, but also requires a total processing time of approximately 34 min, thus fulfilling the rapid contour modification requirements. The initial surface shape of the ϕ100 mm fused silica mirror is presented in Figure 10a, with a surface shape error of RMS 58.006 nm. The surface shape after the first processing (P1) is shown in Figure 10b, with the surface shape error reduced to RMS 17.580 nm, achieving a convergence rate of 69.7%. The surface shape after the second processing (P2) is illustrated in Figure 10c, with the surface shape accuracy further converging to RMS 11.143 nm, and a convergence rate of 36.6%. The total surface shape convergence rate after two modifications reached 80.8%.

The Power Spectral Density (PSD) curves of the surface shape, after the removal of translation and inclination, are presented in Figure 10e for both pre- and post-processing conditions. Notably, the low-frequency roughness (LFR, 0.01 mm^−1^ < f < 0.02 mm^−1^) exhibited significant improvement across both processing stages, decreasing from a root mean square (RMS) value of 5.177 nm to 1.477 nm, resulting in a total reduction of 10.89 dB. The mid-spatial frequency roughness (MSFR, 1 mm^−1^ < f < 0.02 mm^−1^) showed substantial convergence during the first processing stage. In the second processing stage, due to the limited convergence ratio of the specified surface shape, only a few micro-convex peaks were trimmed. Following both processing stages, the intermediate frequency plane type error was reduced from an RMS value of 3.612 nm to 2.473 nm, yielding a total reduction of 3.29 dB. The variation in surface roughness during the modification process is illustrated in Figure 10f. The surface roughness increased from an initial Ra of 0.487 nm to an Ra of 0.974 after the first modification. After the second modification, efficient and high-quality shaping of the workpiece surface was achieved, with surface roughness controlled at Ra 1.719 nm. No significant polymer deposition occurred, providing a solid foundation for the subsequent roughness reduction through CCOS.

### 3.3. Mechanical Properties of Deposition Layer

The mechanical properties of the PDL and the surface of fused silica, processed by ICP single-point for 20 s, were evaluated using a nanoindentation instrument. Loading and unloading experiments were performed on both regions, with six points tested in each. The overall thickness of the polymer deposition layer, being less than 40 nm and exhibiting insufficient uniformity, complicates the accurate measurement of its hardness and elastic modulus. Specifically, the hardness of the PDL is low, necessitating that the indentation depth be limited to one-tenth of the total thickness of the deposition layer to mitigate substrate influence. However, during actual experiments, the probe often penetrates the PDL layer, indenting into the fused silica substrate.

**Figure 11:**
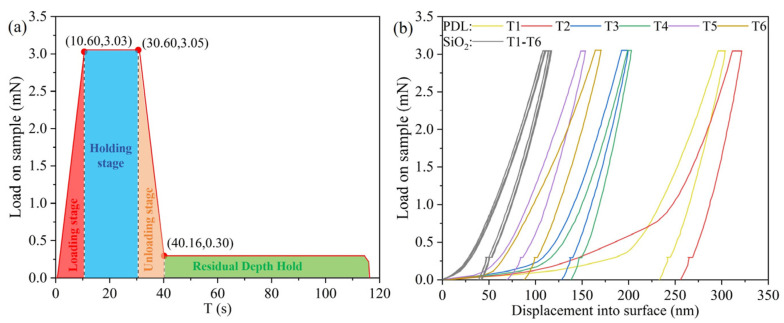
Nanoindentation experiments on PDL areas and fused silica substrates. (**a**) Indentation load function; (**b**) Loading–unloading experimental results.

Nevertheless, certain characteristics of the polymer deposition layer can still be inferred from the surface loading–unloading curve. The load function is illustrated in Figure 11a, with a maximum load of 3000 μN and a holding time of 20 s prior to unloading. The loading and unloading curves for both regions are depicted in Figure 11b. From the perspective of the dispersion of the loading and unloading curves, the results for the PDL region exhibit significantly greater variability, with the indentation depths at T1 and T2 in the PDL region notably exceeding those of the other four measurements. This discrepancy may be attributed to the indentation positions corresponding to case 2/case 3, as shown in Figure 2, indicating that the uniformity of the PDL is inferior to that of the fused silica substrates. Furthermore, regarding overall indentation depth, under identical loads, the indentation depth of the PDL is considerably greater than that of the fused silica. This suggests that as the indentation depth increases, the indenter readily penetrates the PDL, indicating that the hardness of the PDL is significantly lower than that of the fused silica. Additionally, the area enclosed by the loading and unloading curve of the PDL is larger, and the displacement is more pronounced under the same load. This observation implies that the polymer deposition undergoes substantial plastic deformation during loading, making it more susceptible to bending or compression under external forces, with minimal elastic recovery post-unloading. Essentially, this behavior is predominantly plastic. Due to the significant differences in elastic–plastic behavior and hardness between the PDL and the fused silica substrate, abrupt changes in interface stress occur, rendering the PDL prone to detachment and removal under external forces. This further substantiates that during the processing of small tools, when abrasive particles driven by polishing pads act on both the PDL and fused silica substrates under identical loads, the PDL is preferentially removed. By controlling the process parameters, complete removal of the PDL can be achieved, facilitating conformal polishing.

### 3.4. Experiment on Removing PDL by CCOS

The CCOS process is employed to remove the surface PDL following ICP precision modification, due to its significant effectiveness in eliminating medium-frequency and high-frequency errors of optical surfaces and reducing surface roughness. As discussed in Section 3.3, we analyzed the mechanical properties of the PDL and concluded that during CCOS processing, the PDL is preferentially removed over the base material. Conformal polishing can be achieved by appropriately setting the process parameters. Through experimental exploration, the process parameters of CCOS are primarily categorized into the composition of the polishing liquid and the specifications of the processing equipment. Table 5 presents the parameters of the polishing liquid. The abrasive particles consist of 20 nm CeO_2_, which have an exceptionally small particle size, as the thickness of the PDL in actual ICP processing is generally less than 20 nm (Figure 14b). Larger abrasive particles can puncture the deposited layer under pressure and directly impact the fused silica substrate, thereby diminishing the buffering effect of the PDL. Furthermore, smaller abrasive particles can adjust the surface roughness at a higher frequency, facilitating the production of ultra-smooth surfaces of superior quality. To enhance the fluidity and dispersion of abrasive particles in the polishing liquid, DBSA is employed as a liquid lubricant and dispersant. During the application of the polishing liquid, ultrasonic vibration at a frequency of 20 kHz and a power of 400 W is utilized to prevent the agglomeration of abrasive particles and to improve the stability of material removal. The optimal grinding slurry consists of 4.0 wt% CeO_2_, 0.2 wt% DBSA, with the remainder comprising pH regulators and deionized water.

The process parameters of the processing equipment are illustrated in Table 6. Notably, specific selections have been made regarding the type of polishing pad. Due to the relatively low hardness of the asphalt polishing pad, the Softening Point is often used to characterize its hardness. In this study, asphalt with a melting point of 52–55 °C is utilized, which is relatively soft. This characteristic allows the abrasive particles to embed more easily into the asphalt surface during the three-body friction process, thereby reducing the likelihood of abrasive particles scratching the surface. Prior to each experiment, all samples were ultrasonically cleaned in an anhydrous ethanol bath for a minimum of 10 min and subsequently dried with nitrogen. After each processing cycle, the samples were ultrasonically washed with isopropyl alcohol and anhydrous ethanol for 10 min to remove residues, followed by drying with nitrogen.

**Figure 12:**
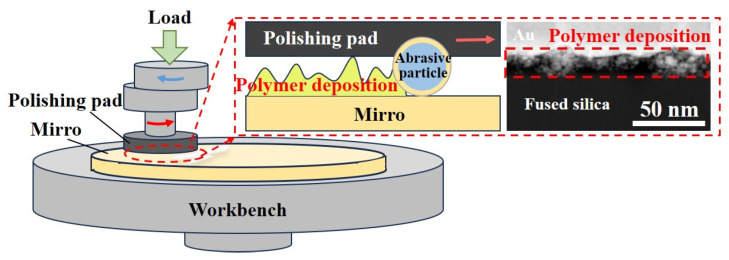
Schematic diagram of CCOS removing polymer deposits.

**Figure 13:**
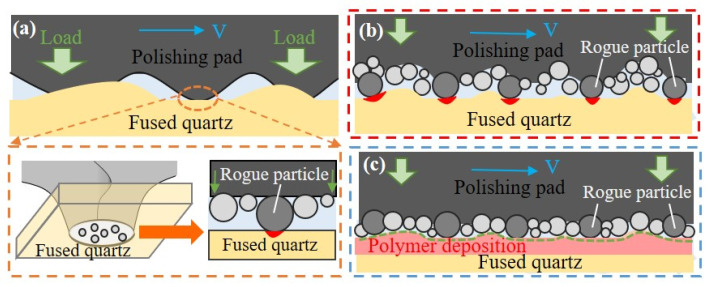
Mechanism of the protective effect of the PDL on the workpiece surface during the CCOS process: (**a**) Microscopic schematic diagram of the contact process between the polishing pad, abrasive particles and the workpiece surface; (**b**) Process of large abrasive particles bearing most of the external load and damaging the workpiece surface; (**c**) Mechanism of buffering and protecting the scratching effect of the PDL on the abrasive particles.

The aforementioned process parameters can effectively eliminate the PDL and enhance processing quality. The processing principle is illustrated in Figure 12. The external load is transferred to the abrasive particles via the mechanical structure of the polishing pad and acts on the workpiece surface through the revolution and rotation of the pad. Due to the significant difference in elastic modulus between the PDL and the fused silica substrate, interfacial stress changes abruptly. Additionally, the hardness of the PDL is much lower than that of the fused silica substrate, facilitating the preferential removal of the PDL under the action of abrasive particles. Moreover, since both the surface of the asphalt polishing pad and the workpiece surface are not smooth or flat, in the initial contact state, the external load is primarily borne by the rough peaks in contact, as depicted in Figure 13a,b. Due to the gradient in the particle size distribution of the abrasive grains, the larger grains bear the majority of the load and act on the workpiece surface. This can lead to surface defects such as pitting and scratches. The PDL exists between the molten silicon substrate and the abrasive particles, which is susceptible to plastic deformation. During the energy transfer process involving the abrasive particles, a portion of the energy is transferred to the PDL, thereby serving a buffering function in the friction process. As illustrated in Figure 13c, the PDL provides a buffering effect for the larger abrasive particles initially embedded in the asphalt polishing pad. This buffering allows the larger abrasive particles to be pressed into the asphalt polishing pad until they share the external pressure with the majority of the abrasive particles, subsequently acting on the fused silica substrate. This mechanism protects the fused silica substrate from surface defects, such as scratches and pits, thereby facilitating the attainment of a non-destructive ultra-smooth surface.

**Figure 14:**
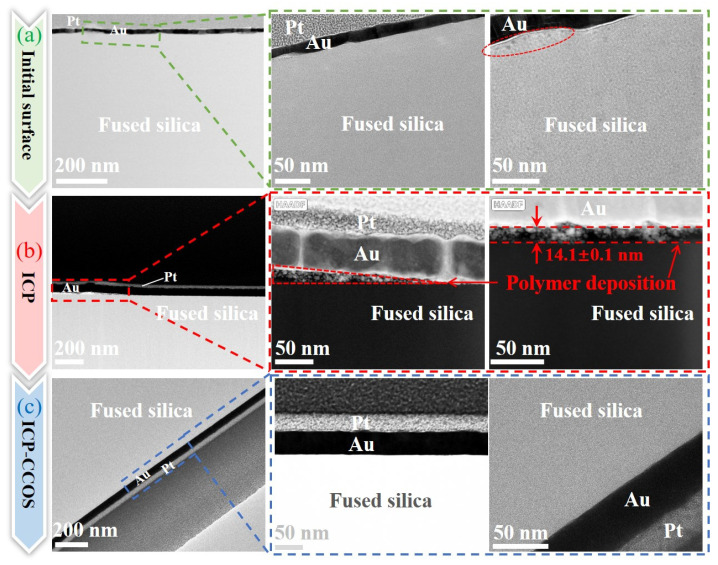
TEM test results of surface and subsurface states during ICP-CCOS processing. (**a**) initial surface; (**b**) surface after ICP processing, with polymer deposition; (**c**) surface after ICP-CCOS processing.

To further investigate the surface and subsurface states during ICP-CCOS processing, TEM was employed to examine and analyze the initial surface, the surface after ICP processing, and the surface after ICP-CCOS. The initial surface is depicted in Figure 14a. Due to surface defects and roughness, the uniformity of the gold plating layer is poor. Upon increasing the magnification to examine the interface of the fused silica, it is observed that although there are undulations at the interface, these undulations correspond to the imaging characteristics of the substrate and represent surface and interface undulations of the fused silica substrate, according to the detection results. After ICP processing, as shown in Figure 14b, the gold plating layer exhibits uniform thickness, with no apparent damage observed on the surface or subsurface of the fused silica. However, a distinct PDL is observed at the interface between the fused silica and the gold plating layer. Upon increasing the magnification, the observed morphology of the Polishing Debris Layer (PDL) reveals loose, porous, coating-like structures when compared to the fused silica substrate, with an average thickness of 14.1 ± 0.1 nm. This aligns with the mechanical properties described in Section 3.3, where the PDL exhibits lower hardness and elastic modulus, and the displacement under the same load is significantly greater than that of the fused silica.

**Figure 15:**
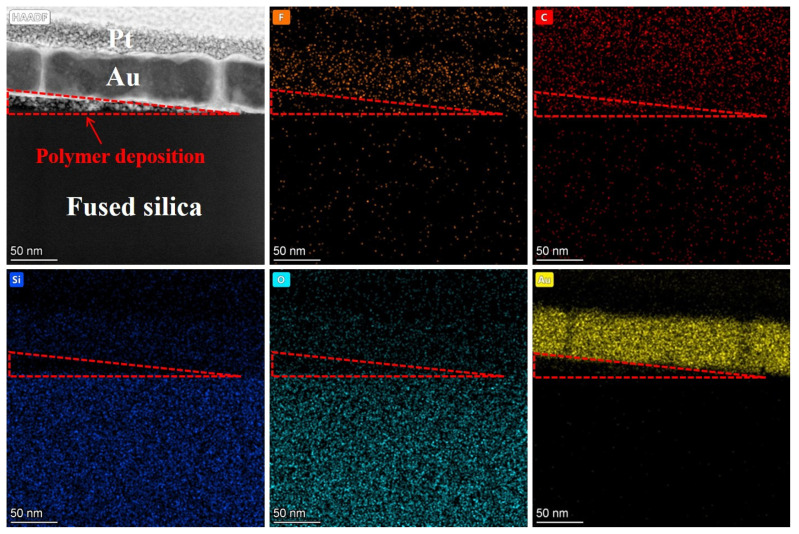
EDS spectrum of PDL area.

**Figure 16:**
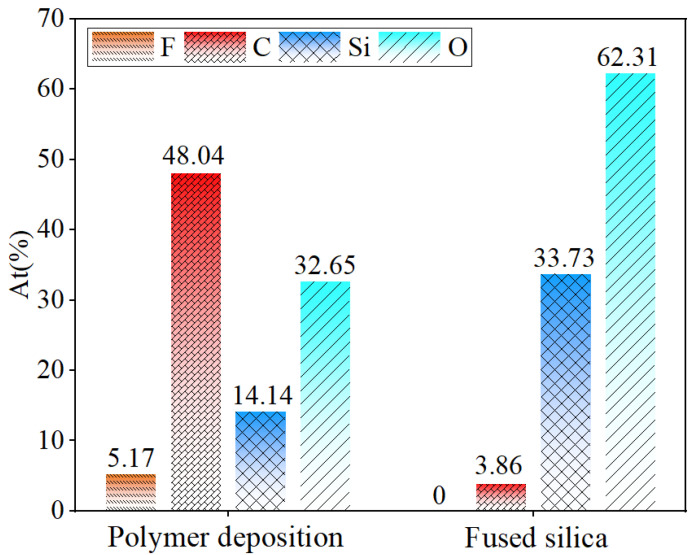
Comparison of each element content between PDL area and fused silica substrate.

To further investigate the characteristics of the PDL, EDS spectral analysis was performed on this region, as shown in Figure 15. Based on the detection results, the triangular PDL area mainly contains Si, O, F, and C elements, while the substrate region does not contain F. The F element content in the triangular region is 5.17%, as shown in Figure 16, consistent with the elements of intermediate products (CF-CF_n_) produced by the reaction between plasma and fused silica. Additionally, the C element content in the polymer deposition area significantly increases to 48.04%. Combined with the C-F bonds in the XPS high-resolution spectrum shown in Figure 5b, it was further confirmed that the F element in the polymer deposition primarily exists in the form of fluorocarbons. An interesting observation is that the O-to-Si ratio in the fused silica matrix is 1.85, while in the deposition area, it is 2.31. This indicates that the O content in the polymer within the deposition area is higher than in the matrix. This also indirectly verifies the presence of MSiO_3_ and metal oxide. An analysis of the characteristic aggregation of elements in the deposition zone into multiple tiny clusters indicates that, compared to the substrate, the composition of the deposition zone is relatively loose. Furthermore, gold atoms exhibit significant penetration into the pores of this area, which aligns with the results of nanoindentation presented in Section 3.3.

**Figure 17:**
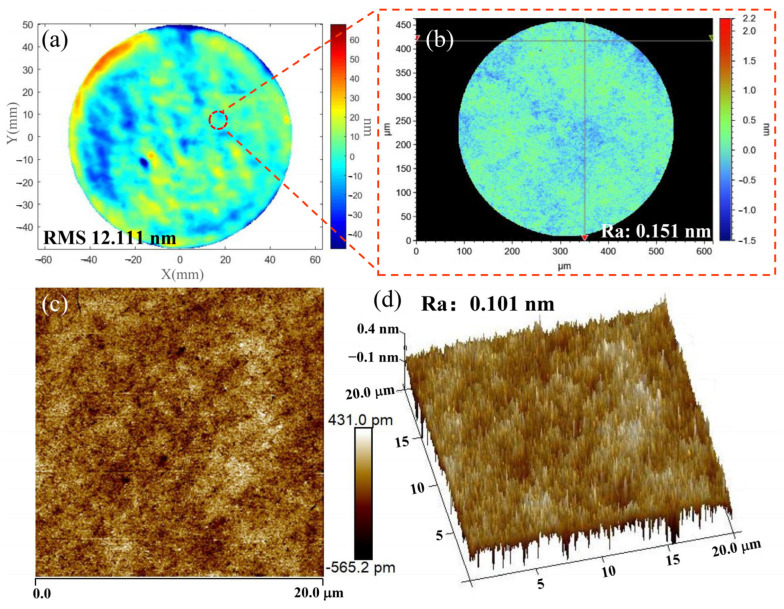
Surface shape and roughness test results of the back surface of ICP-CCOS: (**a**) Surface shape of the back surface of ICP-CCOS; (**b**) Surface roughness after ICP-CCOS; (**c**) 2D results of AFM test; (**d**) 3D results of AFM test.

**Figure 18:**
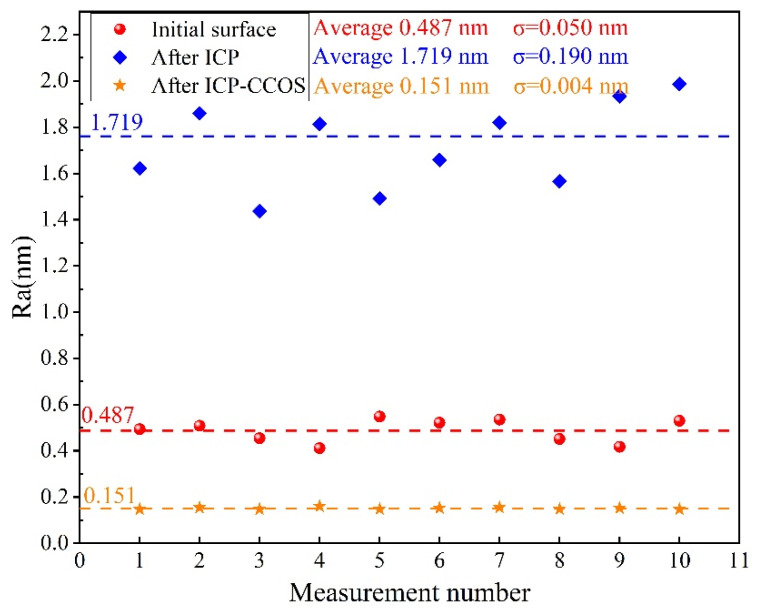
Change results of surface roughness during ICP-CCOS process.

In conclusion, the surface processed by ICP-CCOS is shown in Figure 14c. The thickness of the gold plating layer on the surface is uniform, and the interface with the fused silica is clearly defined. There is no polymer deposition, indicating that CCOS can effectively remove the polymer deposition produced by ICP, and there is no significant damage to the surface or sub-surface of the workpiece, maintaining the damaged-free surface obtained by ICP. The detection results of the accuracy of the surface shape and the roughness after ICP-CCOS processing are shown in Figure 17a,b. The surface shape error is 12.111 nm RMS, and the surface roughness is Ra 0.151 nm. For a more comprehensive evaluation of the surface roughness, AFM was used to detect the sample surface, as shown in Figure 17c,d. The surface roughness measured within the range of 20 µm × 20 µm is Ra 0.101 nm. Due to differences in detection principle, resolution, and detection range between AFM and the white light interferometer, differences in measurement results are inevitable. However, the detection results of both instruments provide insights into the surface roughness. In this paper, the white light interferometer detection results with larger detection range are temporarily taken as the roughness processing quality evaluation index of the ICP-CCOS composite process. The surface shape accuracy improved by 79.1% compared to the initial surface, and the roughness improved by 69.0% compared to the initial surface. The variation in surface roughness during the entire processing procedure is shown in Figure 18. After each stage of processing, 10 positions were randomly measured, and the average value and standard deviation were calculated. The PDL produced after ICP processing was uneven, leading to significant fluctuations in the surface roughness measurement value. The standard deviation increased from 0.050 nm to 0.190 nm.

**Figure 19:**
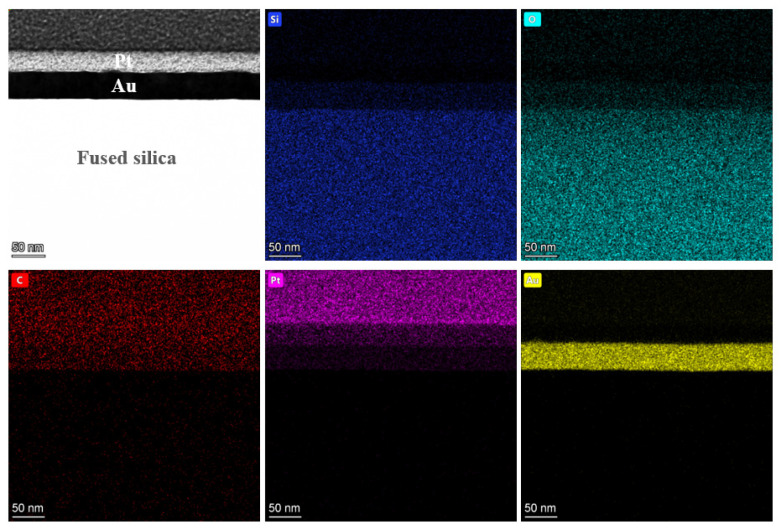
EDS spectrum of final surface after ICP-CCOS.

After processing with ICP-CCOS, the average surface roughness decreased significantly to Ra 0.151 nm within 90 min, and the standard deviation decreased to 0.004 nm, indicating excellent uniformity of surface roughness. An additional EDS energy spectrum analysis was conducted on the area processed by ICP-CCOS. As shown in Figure 19, the thickness of the gold atom layer coated on the surface was uniform, and the boundary between it and the surface of the fused silica was distinct without any overlapping or blurred regions. These findings confirmed that the interface of the processed fused silica was uniform and dense, and there were no defects such as cracks or pitting that led to the infiltration of gold atoms. Furthermore, no enrichment of F elements directly associated with polymer deposition was detected in the interface area, nor was the Ce element from the cerium oxide utilized by CCOS observed. This further confirms that CCOS can effectively remove PDL from the surface after ICP treatment, without the transfer or contamination of Ce elements on the surface.

**Figure 20:**
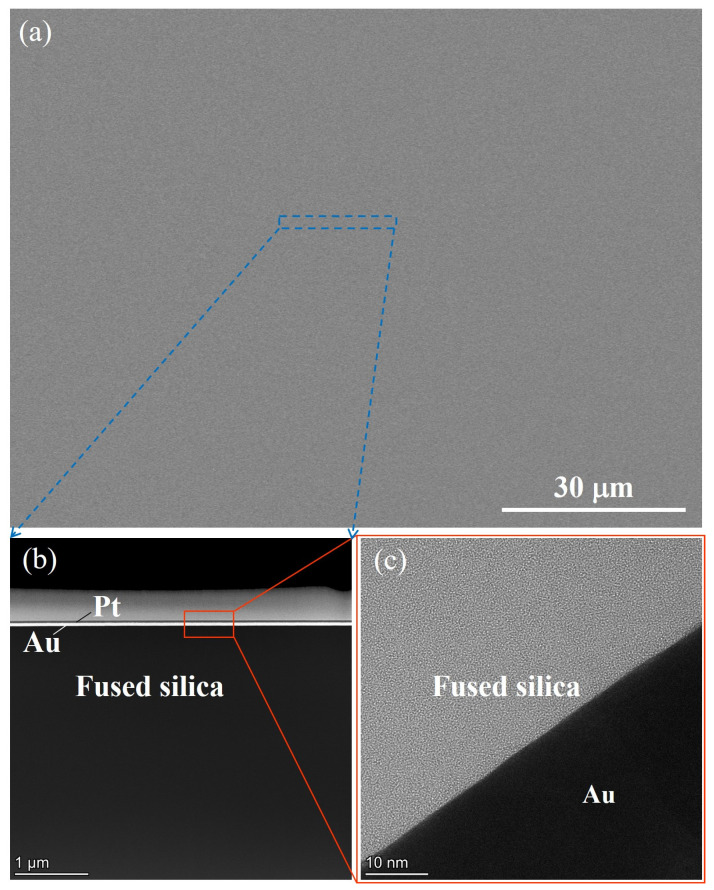
ICP-CCOS fabrication of damage-free ultra-smooth surfaces. (**a**) The uniform surface detected by SEM; (**b**) The detection results of TEM at low magnification; (**c**) The detection results of TEM at high magnification.

The results of the large-scale SEM inspection of the surface following CCOS processing are presented in Figure 20a. The surface exhibits a uniform and excellent quality, with no observable agglomeration, residue, or embedding of cerium oxide abrasive grains. In addition, since the substrate treated by ICP in the previous process (Figure 14b) no longer has subsurface damage, and considering that the CCOS process adopts the same residence time at all points, it means that the processing conditions of the entire surface are consistent. Therefore, we prepared a TEM thin sheet at a random position on the sample. Increasing the magnification of the TEM and carefully observing the surface and subsurface conditions of the fused silica, as shown in Figure 20b,c, revealed no significant damage layers or defects on the surface and subsurface. Moreover, the interface between the fused silica and the gold plating layer was distinct, and no evidence of shadows resulting from subsurface defects was found. This indicates that ICP-CCOS can effectively remove the PDL without introducing new damage, thus preserving the non-destructive surface obtained by ICP and further reducing the surface roughness. In conclusion, by using the ICP-CCOS method, simultaneous improvement of the surface shape accuracy and roughness of the fused silica surface can be achieved, and a high-precision, non-damaging ultra-smooth surface can be efficiently obtained.

## 4. Conclusions

Addressing the issue that the PDL generated during the ICP shaping process affects the final processing accuracy and surface quality of fused silica optical elements, this study thoroughly investigates the causes, elemental composition, and state evolution of the PDL over the dwell time, and it proposes a combined processing method of plasma shaping and small tool polishing (ICP-CCOS). By leveraging the efficient shape modification ability of ICP and the shape preservation and roughness reduction advantages of CCOS, this method breaks the conventional cycle of surface shape modification and roughness convergence, facilitating the rapid convergence of surface shape error and roughness, and resulting in a damage-free, ultra-smooth surface. The main innovative aspects include:

(1) The formation mechanism, chemical composition, and mechanical properties of the PDL produced by plasma processing are analyzed, providing a foundation for the proposed method of the gradient division of dwell time for surface shape trimming, which aims to suppress the deterioration of surface roughness.
(2) Utilizing the low hardness and elastic modulus of the PDL layer formed on the surface after ICP modification, the surface is protected from scratches caused by large abrasive particles during the initial stage of CCOS processing. This helps maintain the damage-free surface obtained after ICP modification and mitigates the limitations of ICP processing, such as roughness deterioration caused by polymer deposition.
(3) Applying the ICP-CCOS method with optimized process parameters eliminates the need for multiple cycles of shape modification and roughness reduction. This method efficiently achieves the simultaneous convergence of surface shape error and roughness, resulting in an ultra-smooth surface with high shape accuracy, exceptionally low roughness, and no damage.

High-performance processing verification for a fused silica mirror with a diameter of 100 mm was carried out using the ICP-CCOS method. Over a period of 124 min, the surface accuracy of the workpiece improved from RMS 58.006 nm to RMS 12.111 nm. Simultaneously, the surface roughness decreased from the deteriorated Ra 1.719 nm to Ra 0.151 nm. The experimental results confirm the feasibility of the ICP-CCOS composite processing method, which can efficiently obtain high-precision and non-damaging fused silica surfaces, offering a novel strategy for the ultra-precision manufacturing of fused silica mirrors and providing important technical support for the wide application of high-quality optical components in fields with extreme performance requirements.

## Figures and Tables

**Table 1:** ICP process parameters with polymer deposition.

Power (W)	Gas Composition	CF_4_ Flow Rate (sccm)	Ar Flow Rate (sccm)	Processing Distance (mm)	Point Dwell Time(s)
350	Ar, CF_4_	20.00	800.00	17.0	20

**Table 2:** ICP process parameters with equal dwell time intervals.

Power (W)	Gas Composition	CF_4_ Flow Rate (sccm)	Ar Flow Rate (sccm)	Processing Distance (mm)	Point Dwell Time (s)
350	Ar, CF_4_	20.00	800.00	17.0	5 s apart

**Table 3:** The parameters of ICP single-point multiple cumulative processing and single continuous processing.

Number	Power (W)	Gas Composition	CF_4_ Flow Rate (sccm)	Ar Flow Rate (sccm)	Processing Distance (mm)	Residence Mode (s)
A	300	Ar, CF_4_	20.00	800.00	17.0	Process 5 times, 2 s each time
B	300	Ar, CF_4_	20.00	800.00	17.0	Process once for 10 s

**Table 4:** Process parameters of the resident time gradient partitioning method.

Number	Power (W)	Gas Composition	CF_4_ Flow Rate (sccm)	Ar Flow Rate (sccm)	Processing Distance (mm)	Shape Convergence Rate(%)	Processing Time (min)
P1	300	Ar, CF_4_	20.00	800.00	17.0	60	26.7
P2	300	Ar, CF_4_	20.00	800.00	17.0	20	6.6

**Table 5:** The composition of polishing slurry.

Abrasive Particle	Particle Size (nm)	PH	Additive	Ultrasonic Vibration Frequency (Hz)	Ultrasonic Vibration Power (W)	Temperature (°C)
4.0 wt% CeO_2_	20	8.0	0.2 wt% DBSA	20k	400	25–27

**Table 6:** Process parameters of CCOS equipment.

Pressure (N)	Rotational Speed (rad/min)	Orbital Speed (rad/min)	Eccentric Distance (mm)	Polishing Time (min)	Polishing Pad Diameter (mm)	Polishing Pad Material	Softening Point of Asphalt (°C)
4	50	110	3.0	90	20.0	Asphalt	52–55

## Data Availability

The data presented in this study are available on request from the corresponding author.
